# Single Particle Electron Microscopy Analysis of the Bovine Anion Exchanger 1 Reveals a Flexible Linker Connecting the Cytoplasmic and Membrane Domains

**DOI:** 10.1371/journal.pone.0055408

**Published:** 2013-02-05

**Authors:** Jiansen Jiang, Nathaniel Magilnick, Kirill Tsirulnikov, Natalia Abuladze, Ivo Atanasov, Peng Ge, Mohandas Narla, Alexander Pushkin, Z. Hong Zhou, Ira Kurtz

**Affiliations:** 1 Department of Microbiology, Immunology and Molecular Genetics, and California NanoSystems Institute, University of California Los Angeles, Los Angeles, California, United States of America; 2 Structural Computational Biology and Molecular Biophysics Program, Baylor College of Medicine, Houston, Texas, United States of America; 3 Department of Medicine, D. Geffen School of Medicine at University of California Los Angeles, Los Angeles, California, United States of America; 4 New York Blood Centre, New York, New York, United States of America; 5 Brain Research Institute, University of California Los Angeles, Los Angeles, California, United States of America; Emory University School of Medicine, United States of America

## Abstract

Anion exchanger 1 (AE1) is the major erythrocyte membrane protein that mediates chloride/bicarbonate exchange across the erythrocyte membrane facilitating CO_2_ transport by the blood, and anchors the plasma membrane to the spectrin-based cytoskeleton. This multi-protein cytoskeletal complex plays an important role in erythrocyte elasticity and membrane stability. An in-frame AE1 deletion of nine amino acids in the cytoplasmic domain in a proximity to the membrane domain results in a marked increase in membrane rigidity and ovalocytic red cells in the disease Southeast Asian Ovalocytosis (SAO). We hypothesized that AE1 has a flexible region connecting the cytoplasmic and membrane domains, which is partially deleted in SAO, thus causing the loss of erythrocyte elasticity. To explore this hypothesis, we developed a new non-denaturing method of AE1 purification from bovine erythrocyte membranes. A three-dimensional (3D) structure of bovine AE1 at 2.4 nm resolution was obtained by negative staining electron microscopy, orthogonal tilt reconstruction and single particle analysis. The cytoplasmic and membrane domains are connected by two parallel linkers. Image classification demonstrated substantial flexibility in the linker region. We propose a mechanism whereby flexibility of the linker region plays a critical role in regulating red cell elasticity.

## Introduction

AE1 is a major membrane protein in erythrocytes (25 to 30% of the total membrane mass) that mediates anion exchange and participates in control of the erythrocyte shape [1], [2], [3], [4], [5], [6]. AE1 also participates in the formation of a senescent cell antigen in aged erythrocytes [7]. The structure of AE1 is likely altered in several pathological conditions including thalassemia, sickle cell anemia, and red cells harboring the malaria parasite *Plasmodium falciparum* [8], [9], [10], [11], [12], [13]. In the erythrocyte membrane, AE1 forms oligomers that are predominantly dimers and tetramers. The AE1 monomer (∼100 kDa) consists of an ∼45 kDa N-terminal cytoplasmic domain and an ∼53 kDa C-terminal membrane domain [1], [2]. The C-terminal membrane domain is involved in mediating anion exchange. The N-terminal cytoplasmic domain provides binding sites for many proteins, including ankyrin, band 4.2 and band 4.1 proteins, glycolytic enzymes, hemoglobin, deoxyhemoglobin, hemichromes, p72syk protein tyrosine kinase, adducin and integrin-linked kinase [2], [3], [6], [14], [15]. Glycolytic enzymes, hemichromes and deoxyhemoglobin bind to the negatively charged extreme N-terminus of AE1 via electrostatic interactions. AE1 connects the spectrin-based cytoskeleton to lipid bilayer via ankyrin [1], [2], [3], [15] and adducing [6], [16]. It is widely accepted that interactions between AE1 and the membrane cytoskeleton play a role in mediating erythrocyte shape control [2], [3], [14], [15], [16]. Quantitative deficiency of AE1 resulting in decreased anchoring of the lipid bilayer to the membrane cytoskeleton leads to loss of membrane cohesion and resultant membrane surface area loss and generation of spherocytic red cells in hereditary spherocytosis [17]. On the other hand, a qualitative defect resulting in an in-frame deletion of nine amino acids in the cytoplasmic domain of AE1 [2], [18], results in a marked increase in membrane rigidity and ovalocytic red cells in Southeast Asian Ovalocytosis (SAO).

Since AE1 is a highly abundant membrane protein with a million copies in each red cell and pure plasma membrane can be readily obtained from enucleated red cells, it should be one of the very few membrane proteins that can be directly purified from natural membranes. However, the high heterogeneity of natural AE1 because of various covalent modifications, the presence of different oligomeric forms and complexes with other erythrocyte proteins has made it very difficult to purify in a highly homogeneous form for structural studies. In addition, treatment with chaotropic agents and non-physiological alkaline pH used in the separation of accessory proteins from AE1 can significantly change its native structure [1], [19], [20], [21]. Indeed, an earlier electron microscopy (EM) study of negatively stained AE1 purified from KI/EDTA stripped human erythrocyte membranes revealed a high level of heterogeneity in size and shape [22]. The calculated average mass of AE1-detergent micelles in octyl-POE and C_12_E_8_ was ∼1,500 and ∼500 kDa, respectively. Despite its heterogeneity, octyl-polyoxyethylene solubilized AE1 formed two-dimensional (2D) arrays (unit cell dimensions: a = b = ∼11 nm) in the presence of dimyristoyl phosphatidylcholine [22]. Each unit cell contained three elongated densities hypothesized to be three AE1 dimers, but the membrane and cytoplasmic domains were not resolved.

Additional attempts to determine the structure of AE1 have involved separately studying the membrane and cytoplasmic domains. The membrane domain generated by trypsin digestion of purified human AE1 was reconstituted into 2D sheets with lipids [19]. Electron crystallography of negatively stained 2D crystals revealed a U-shaped 6×11 nm structure with a thickness of 8 nm [20]. Recently, 2D crystals of the human AE1 membrane domain, generated by trypsin digestion of erythrocyte membranes and trihalose-embedding, were used to generate a 7.5 Å resolution structure by electron crystallography [23]. However, the structure revealed only 7 of the expected 14 transmembrane helices, likely because the alkaline treatment with 0.1 M NaOH used for stripping of accessory proteins from AE1 had significantly modified the topology of the membrane domain [24]. A 3D crystal of the membrane domain of the human AE1 was first reported in 2002, but it only diffracted X-rays to ∼14 Å and was insufficient for structural determination [21]. The 3D structure of the cytoplasmic domain (aa. 1–379) of human AE1 was solved by X-ray crystallography to 2.6 Å resolution [14]. The structure revealed a rectangular prism-shaped symmetrical dimer stabilized by interlocking arms. Residues 1–54, 202–211 and 357–379 were not visible in this crystal structure, suggesting their flexibility. Because none of these structures provides the spatial organization of full-length AE1, it remains unclear whether the cytoplasmic domain is tightly linked to membrane domain, or whether it can move relative to the membrane domain as has been proposed [18]. Such elasticity could be mediated by a flexible linker region between the cytoplasmic and membrane domains. Although it is currently impossible to precisely localize the linker area, it likely involves aa. 357–408. The selection of the first amino acid residue (aa. 357) is based on the flexibility of aa. 357–379 in the crystallography structure of the cytoplasmiic domain [14]. The location of the last residue (aa. 408) is selected on the basis of current topology models predicting the first transmembrane segment in the membrane domain starting from aa. 401 or 409 [1], [2], [3], [4], [5], [6]. The SAO mutation (aa. 400–408) resulting in a marked increase in membrane rigidity and ovalocytic red cells also suggests that aa. 400–408 are located in the C-terminal part of the linker [18]. In addition, the secondary structure analysis of aa. 357–408 using different prediction models suggests mostly coiled structure for this region.

In the present study, we obtained biochemically homogeneous, native full-length AE1 dimers by using a non-denaturing purification method. By single particle EM reconstruction we show that the AE1 dimer has an elongated structure consisting of a double-humped cytoplasmic domain and an oval-shaped membrane domain tethered by two linkers. Image classification revealed groups of AE1 dimers with different tilt orientations of cytoplasmic domain relative to the membrane domain suggesting flexibility of the two linkers that connect the cytoplasmic domain to the anchored membrane domain. This linker’s flexibility points to a novel pivot mechanism by which AE1 is involved in regulating membrane elasticity, a critical determinant of shape changes necessary for the red cells to transit through capillaries whose dimensions are much smaller than that of the cell.

## Materials and Methods

### Preparation of Erythrocyte Ghosts

Bovine AE1 is not glycosylated [25] and less heterogeneous, and therefore was used in our study. Bovine erythrocyte ghosts were prepared from whole bovine blood (Quad Five) according to Casey and Reithmeier [26] with our modifications in order to preserve native structure of AE1. Fresh defibrinated whole bovine blood was washed 5 times with PBS. This and all following steps were performed at 4°C. Supernatant after centrifugation for 10 min at 3,000 g and a thin top layer of white cells were removed. Red cells were suspended (1∶10) in 5 mM Na phosphate, pH 8.0, containing complete protease inhibitors cocktail (Roche) and 0.2 mM dithiotreitol (DTT). After overnight incubation the suspension was centrifuged for 40 min at 30,000 g. The supernatant was discarded and the upper less dense pellet layer was resuspended in the same buffer containing 0.5 M NaCl and 1 mM EDTA, and then centrifuged again for 40 min at 30,000 g. The operation was repeated up to 10 times until the supernatant was colorless (no hemoglobin is present). At this point only one layer was present in the pellet. The pellet was washed twice in PBS and centrifuged at 30,000 g for 40 min. The final pellet was resuspended in PBS (1∶10) and used directly for AE1 purification or kept frozen at −80°C.

### AE1 Purification

The heterogeneity of natural human AE1 has prevented its purification in a highly homogeneous form required for structural studies. Given that there is significantly lesser heterogeneity of bovine AE1 [25] and a very high sequencing homology (77% identity, 86% homology) between the human and bovine AE1 proteins, we used bovine AE1 instead of human AE1 in the current study. We also excluded alkaline and chaotropic agent treatments of erythrocyte membranes used in previous studies to strip complexed proteins from AE1 that have been shown to significantly change its native structure [1], [27] [24].

The ghost suspension from the previous step was pelleted at 30,000 g for 40 min. The pellet was mixed (1∶20) with 100 mM Tris-HCl, pH 7.5, containing complete protease inhibitors cocktail (Roche) and 2% dodecyl maltoside (DDM, Anatrace). After 2 h incubation, the suspension was centrifuged at 18,000 g for 40 min. The supernatant was loaded onto a 3×5 cm column of DE-52 (Whatman) equilibrated with 50 mM Tris-HCl, containing 0.03% DDM. Proteins were eluted from the column with a linear 0–0.5 M NaCl gradient in the same buffer. AE1 was eluted with 0.3–0.5 M NaCl. The fractions containing AE1 were collected and concentrated to ∼10 mg/ml protein concentration using a Centricon 50 centrifuge filter device (Millipore). The concentrated sample was further subjected to size-exclusion chromatography to obtain the dimeric form of AE1. Approximately 0.3 ml of the concentrated AE1 solution was loaded on a Superose 6™ 10/300 column (GE HealthCare) equilibrated with 50 mM Tris-HCl, pH 7.5, containing 0.03% DDM and 0.15 M NaCl. The fraction containing the AE1 dimer was immediately used for EM grids preparation.

### SDS-PAGE and Immunoblotting

SDS-PAGE was performed using pre-fabricated 7.5% polyacrylamide gels from Bio-Rad [28]. The gels were stained for proteins with Coomassie Blue R (Sigma) or electrotransferred onto PVDF membranes (GE Health Care). Our bovine AE1 specific antibody bAE1-A1 raised in rabbit was used at a 1∶1,000 dilution. A secondary horseradish peroxidase (HP) conjugated mouse anti-rabbit antibody (Jackson Immunoresearch) was used at a dilution 1∶10,000. AE1 bands were visualized using an ECL kit and Hyperfilm ECL (GE Health Care).

### EM Data Collection

Freshly prepared bovine AE1 dimers were negatively stained with 0.8% uranyl formate on grids coated with carbon film. Micrographs were then recorded on a 4k×4k CCD camera at 70,000× magnification using Leginon [29], [30] in an FEI Tecnai F20 electron microscope operated at 200 kV. Two types of images were recorded: tilted for orthogonal tilt reconstruction (OTR) and untilted for in-depth 2D image classification and 3D structure refinement.

For OTR images, the grid was tilted at two orthogonal angles (−45° and +45°) to acquire a pair of micrographs for each specimen area of interest, one micrograph at each tilt angle. In total, 358 sets of tilt pairs were collected.

For 2D image analysis and 3D structure refinement, 1275 micrographs were collected without tilting the grid. In total, 174,197 particles were extracted from the best 669 micrographs. These micrographs have defocus values ranging from −1.0 to −1.8 µm, as determined by CTFIND [31].

### Orthogonal Tilt Reconstruction (OTR) using Tilt Images

For OTR, we obtained two image data sets for each specimen area: a −45° tilt set and a +45° tilt set. Particles in the −45° tilt set were used for classification and particles in the +45° tilt set were used for 3D reconstructions. The following procedure was used to obtain reliable 3D maps without using initial models, thus eliminating possible model bias.

Corresponding particles from tilt pairs were picked out automatically by the *ApTiltPicker.py* program in Appion [32], [33] and verified by manual inspection. 132,517 pairs of particles were selected. The 132,517 particle images in the −45° tilt particle set were classified for 9 iterations with the *refine2d.py* program in EMAN to generate 100 class averages. These class averages were then used as references to align the same particles with SPIDER [34] and subsequently classified these aligned particles into 100 classes using the correspondence analysis method (i.e., *CA S* command) in SPIDER.

All particles in each class of the −45° tilt set have the same feature (*i.e.*, same view), but their corresponding particles in the +45° tilt set represent different views, which we called *ortho-views* of the class. Ortho-views of the same class have different orientation parameters, which were calculated based on the 90° tilt angle difference and the in-plane rotation parameters using a custom script called *combine_ang.bat*. From the ortho-views associated with each class, we obtained one 3D OTR map. Since we had 100 classes in the −45° tilt particles, we obtained a total of 100 OTR maps from the +45° tilt particles. These maps were improved iteratively by using center parameters refined by aligning ortho-views against their projections computed from the 3D OTR maps.

To select the best from these 100 OTR 3D maps, we further evaluated them against class averages of the −45° tilt particle set. In this procedure, for each OTR map, we made one computed projection at orientation (0, 0, 0), which corresponds to the orientation of the associated class average in the −45° tilt particle set. We visually compared this projection and the class average for feature consistency and selected the best 25 OTR maps. These 25 ORT maps then aligned using the *align3d* program in EMAN [35] and averaged.

### 2D Classification and 3D Structure Refinement

The averaged 3D OTR map was used as the starting model to align the 174,197 particles using the projection matching method in EMAN. A 3D map was obtained by combining the aligned particles and iteratively refined. The angular interval between projections was gradually decreased from 15° to 5° during the course of refinement. The map was corrected for the effect of contrast transfer function with phase flipping and imposed with 2-fold symmetry. The resolution of 3D reconstruction was estimated by FSC calculated with the *eotest* program in EMAN. The 3D model was visualized with the UCSF Chimera [36].

To assess the flexibility of cytoplasmic domain, 16,061 front-view particles and 7,644 side-view particles were selected from 174,197 particles based on the result of projection matching. Both the front-view particles and side-view particles are then classified into subgroups.

## Results

### Purification of Bovine AE1

**Figure 1:**
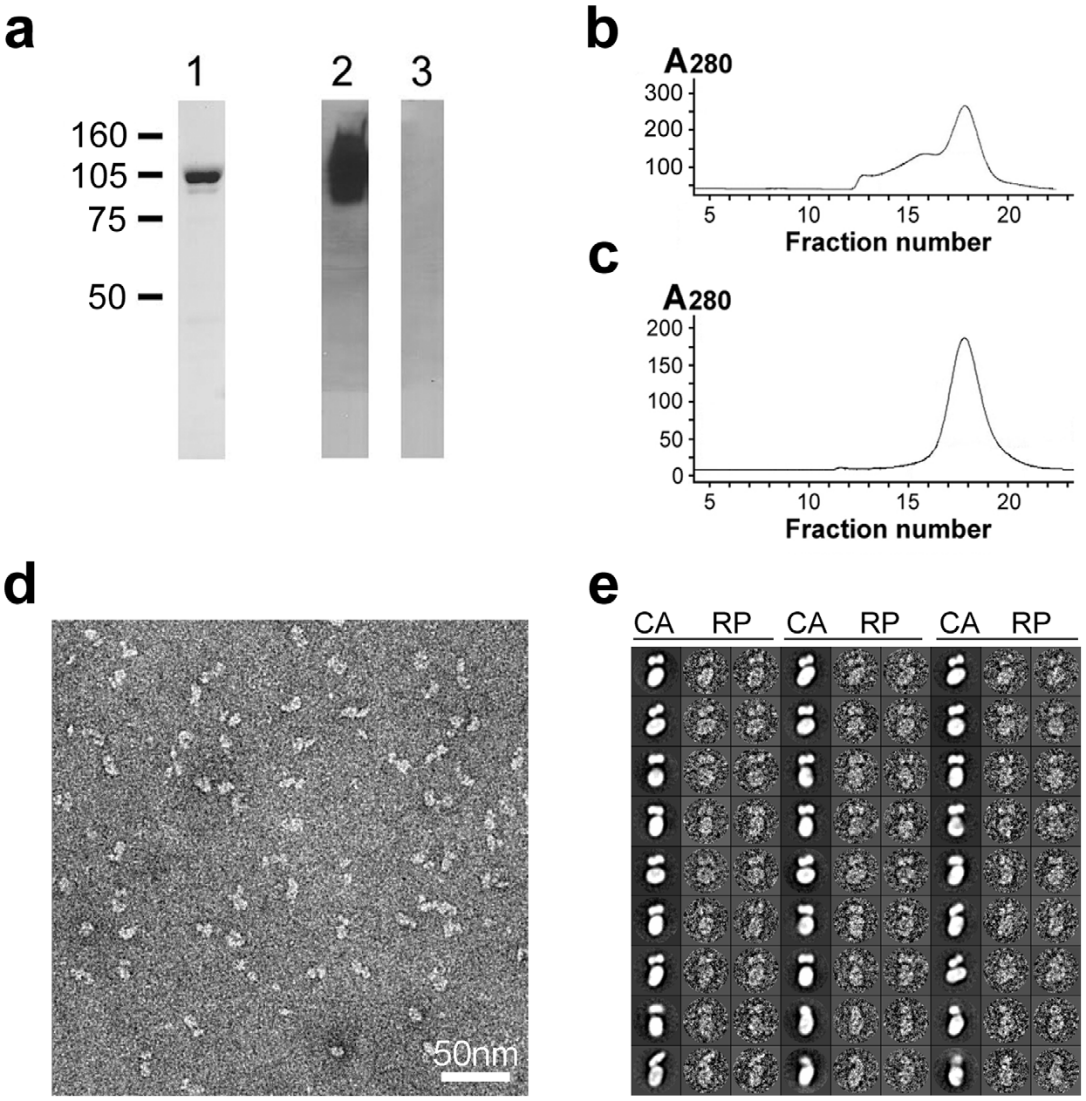
Purification and EM of bovine AE1. (**a**) SDS-PAGE of bovine AE1 after ion-exchange chromatography. Lane 1: Coomassie stained gel; Lanes 2,3: immunoblotting, 2: immune serum; 3: preimmune serum. (**b,c**) Size-exclusion chromatography examination of bovine AE1 sample after the ion-exchange chromatography step (top) and the dimer fraction after the size-exclusion chromatography step (**c**) showing a peak corresponding to dimeric AE1. (**d**) A representative area of transmission EM micrograph of bovine dimeric AE1 stained with 1% uranyl formate. (**e**) Representative class averages (CA) and the corresponding raw particle (RP) images of AE1. Each class average is obtained by averaging about 100 particle images. The side length of each box is 26 nm.

AE1 in the erythrocyte membrane exists either in a free state or as a multi-proteitn complex with other erythrocyte proteins. Previous investigators extracted both pools of AE1, and used alkaline and chaotropic agents to strip off accessory proteins that have been shown to significantly change the native structure of AE1 [1], [24]. In this study, we developed a non-denaturing extraction method to purify free AE1. Our protocol for AE1 purification included several steps to ensure homogeneity of the resulting AE1 preparation: (1) Depletion of erythrocyte membranes (ghosts) from accessory proteins using an optimized buffer at pH 8.0; (2) Ion-exchange chromatography to separate AE1 forms having different covalent modifications; (3) Size-exclusion chromatography to purify AE1 dimers; (4) Use of freshly prepared AE1 dimers for electron microscopy to prevent potential protein oligomerization and aggregation during storage. SDS-PAGE and size-exclusion chromatography showed the homogeneity of our bovine AE1 preparation (Fig. 1a–c).

### 3D Reconstruction of Full-length Dimeric AE1

EM micrograph of negatively stained bovine AE1 particles revealed complexes of different shapes possibly representing AE1 dimers viewed at different orientations (Fig. 1d). The class averages of particle images showed consistent features among these particles (Fig. 1e).

**Figure 2:**
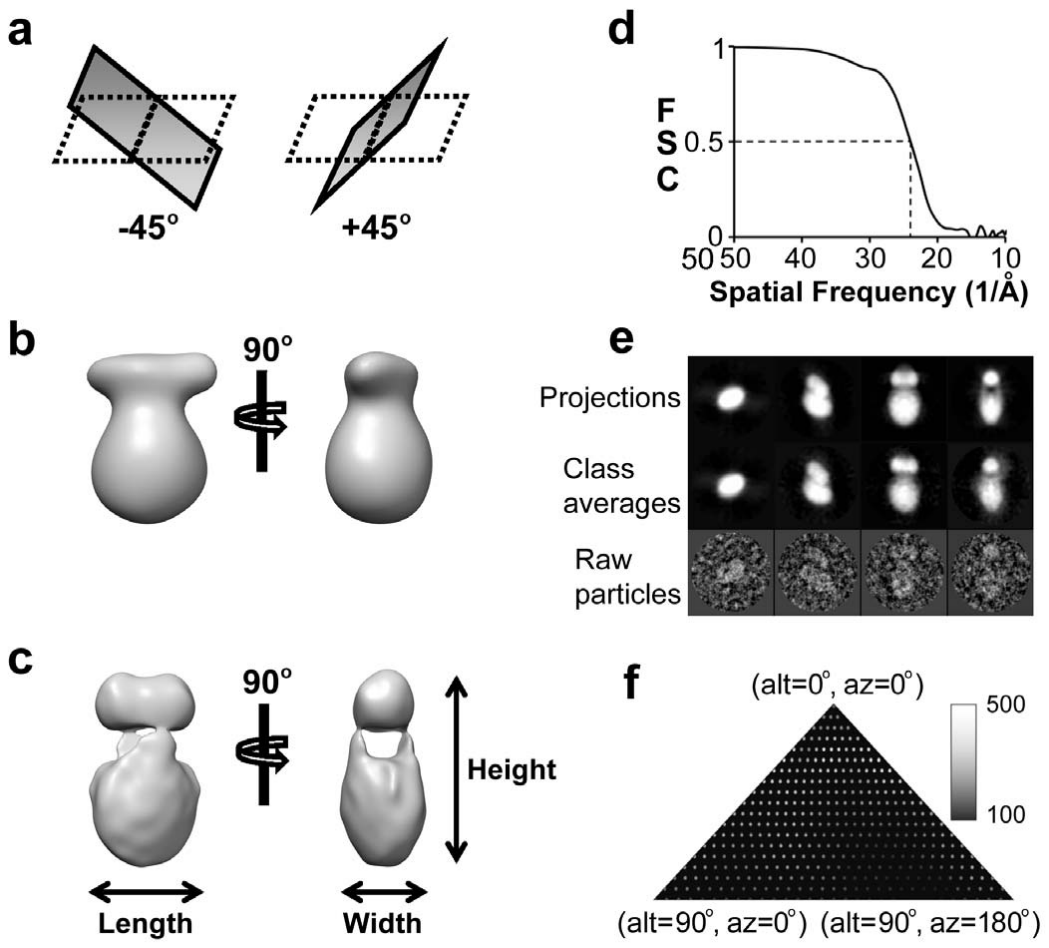
Single-particle reconstruction of bovine AE1. (**a**) Schematic illustration of the OTR data collection method. For each target sample area, two micrographs were recorded with the grid tilted at −45° and +45°, respectively. (**b**) 3D map generated by averaging 25 OTR maps. Two orthogonal views, defined as front view (left panel) and side view (right panel), are shown. (**c**) Final map obtained by merging 174,197 particle images with single particle reconstruction method. The map in (**c**) is shown in the same orientations as in (**b**). (**d**) Fourier shell correlation (FSC) coefficient between two reconstructions obtained from even- and odd-numbered particle images. The effective resolution is estimated to be 2.4 nm using the 0.5 FSC cut-off. (**e**) Comparisons of the computed projection, class average, and raw particle. Four representative views (top, tilt, front, and side) are show from left to right, respectively. (**f**) Euler angle distribution of classified particles. The brightness of each point indicates the number of particles used in the class average in that orientation.

To reconstruct an initial 3D map of AE1 in the absence of a starting model, we used the orthogonal tilt reconstruction (OTR) approach [37], which generated reliable 3D models without having the problem of a missing cone. In total, 358 tilt pairs of micrographs were collected by tilting the EM grid at orthogonal angles (−45° and +45°) for OTR (Fig. 2a). We obtained 100 independent reconstructions, from which 25 consistent ones were selected and further aligned to generate an average 3D model (Fig. 2b).

We obtained an improved reconstruction by combining 174,197 particles of untilted samples by projection matching using the above averaged OTR map as a starting model (Fig. 2c). Since the 3D crystal structure of cytoplasmic domain and the 2D crystal structure of membrane domain are available and been shown to possess 2-fold symmetry, and the 3D model from OTR looks consistent with the symmetry, 2-fold symmetry was then forced throughout the course of structure refinement. The refinement was continued until no further improvement in the structures was observed and the reconstruction resolution of the final 3D model converged at 2.4 nm at 0.5 FSC cut-off (Fig. 2d). The 3D reconstruction was validated by good consistency in the comparisons of the computed projection from the 3D volume with the corresponding class average and raw particles (Fig. 2e). The particles on the carbon support film showed a slightly nonuniform distribution of orientation with a small region of Euler angles containing less particles (Fig. 2f). Since sufficient particle images were used, this issue was negligible to the 3D reconstruction. Even though a large number of particles were used in the 3D reconstruction, the resolution was limited to 2.4 nm due to two possible reasons: (1) the flexible orientation of the cytoplasmic domain (see below), resulting heterogeneous conformations of particles which compromised the resolution when combined to generate a 3D reconstruction; (2) the technical limitation of the negative staining (size of stain microcrystals and penetrability into ultrasturctures), as most of the reported 3D reconstructions from negatively stained samples are limited to a resolution of 2.0 nm [38].

The single-particle reconstruction of the full-length dimeric AE1 has an elongated shape (15×9×6.5 nm) with a small and a larger structure (Fig. 2c). The small structure has a double-humped shape. The large structure has an oval shape and is not separated into two parts likely because bound detergent molecules prevent the penetration of the stain deep into the protein. The small and large portions of the structure are well separated by 3 nm gap crossed with two narrow pillar-like linkers on opposite sides (Fig. 2c).

### Localization of CYTOPLAMIC AND MEMBRANE DOMAINS in the AE1 Dimer

Our antibodies against different parts in the cytoplasmic and membrane domain of bovine AE1 did not work well on immunoelectron microscopy. Therefore we used the published 2.6 Å resolution crystal structure of the human AE1 cytoplasmic domain to localize the cytoplasmic domain in our single-particle reconstruction. Although the crystal structure of the cytoplasmic domain was solved at pH 4.8, site-directed spin labeling studies in combination with conventional electron paramagnetic resonance and double electron resonance spectroscopy performed at neutral pH demonstrated that the structure of the cytoplasmic domain (residues 55–356) is indistinguishable from the crystal structure determined at pH 4.8 [39].

**Figure 3:**
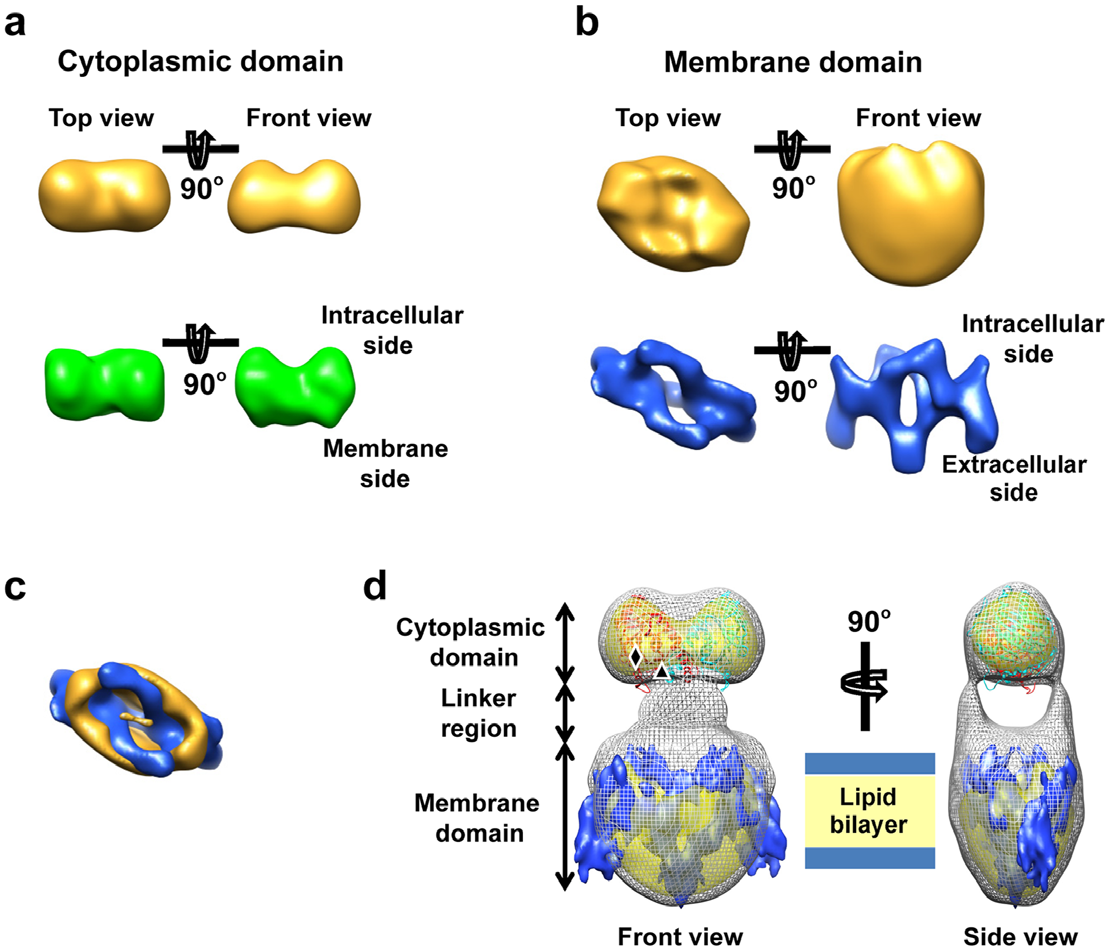
Fitting of the atomic structure of cytoplasmic domain [**14**] **and the 7.5 Å resolution 2D crystal structure of membrane domain** [**23**] **into our 3D map of full length AE1 dimer.** (**a**) Shaded surface views of the atomic structure of cytoplasmic domain (PDB ID: 1HYN) filtered to 2.4 nm resolution (green) compared to the corresponding views of cytoplasmic domain resolved in the EM single-particle reconstruction (gold) of full-length AE1 dimer. In the EM map, the membrane domain of AE1 dimer is removed for clarity. The two structures are similar in size and in having a double-humped shape on their cytoplasmic side. (**b**) Shaded surface views of AE1 membrane domain resolved from 2D crystals embedded in trehalose (EMDB ID: 1645) filtered to 2.4 nm resolution (blue), as compared to the corresponding views of membrane domain resolved in the EM single-particle reconstruction (gold). The extracellular and intracellular sides identified in the published 2D crystal structure were used to define the orientation for comparison. (**c**) Superposition of the two structures of membrane domains described in (**b**) viewed from the cytoplasmic side (top view). The EM single-particle reconstruction is rendered at higher density threshold to show the deep canyon, which is consistent with the membrane domain structure from 2D crystals. (**d**) Fitting the EM single-particle reconstruction of full-length AE1 dimer with the crystal structure of cytoplasmic domain (red and cyan) and 2D crystal structure of membrane domain (blue). The single-particle reconstruction is rendered in two density threshold values: at low threshold (gray mesh) and a high threshold (yellow). The approximate positions of N-terminus and C-terminus of the cytoplasmic domain are labeled with diamond and triangle, respectively.

The dimeric crystal structure of the cytoplasmic domain filtered to 2.4 nm resolution resembles the small end of the EM reconstruction, in the overall size and in having a double-humped shape (Fig. 3a). The ribbon models of the crystal structure of the cytoplasmic domain fit well with the smaller end of our map (Fig. 3d). In this fitting, the C-terminus of the atomic structure of the cytoplasmic domain, which is the N-terminal portion of the full-length AE1, is positioned next to the linker density. We therefore conclude that the small portion resolved in the single-particle reconstruction corresponds to two copies of the cytoplasmic domain. By inference, the membrane domain of AE1 was assigned to the large end of the elongated structure. Indeed, the 2.6 Å resolution crystal structure of the human AE1 cytoplasmic domain did not fit the proposed membrane domain (data not shown). The large end of the elongated structure better fit the 7.5 Å resolution structure of the membrane domain determined by electron crystallography (Fig. 3b). The best fit gave an orientation with the extracellular side of the 2D crystal structure away from the cytoplasmic domain and the intracellular side facing the connector region in our single particle reconstruction model. Both structures have a deep canyon facing the linkers (Fig. 3c). In addition, the canyon-like feature was also observed in the 3D map reconstructed from a negatively stained 2D crystal of the AE1 membrane domain [20]. Flipping the orientation of the fitted 2D crystal structure generated mismatches in many regions, including this canyon. Noticeable differences were identified between our structure and the 2D crystal structure of the membrane domain [23]. First, a small portion of the 2D crystal structure protrudes out from the EM density map (Fig. 3d). Second, the single-particle model has smooth surface without the protruding “spikes” that are present in the 2D crystal structure. These differences are likely due to the alkaline treatment used to deplete accessory proteins from human AE1 to obtain the membrane domain used for 2D crystallization [23] that could have significantly changed/denatured its structure as previously demonstrated [24].

**Figure 4:**
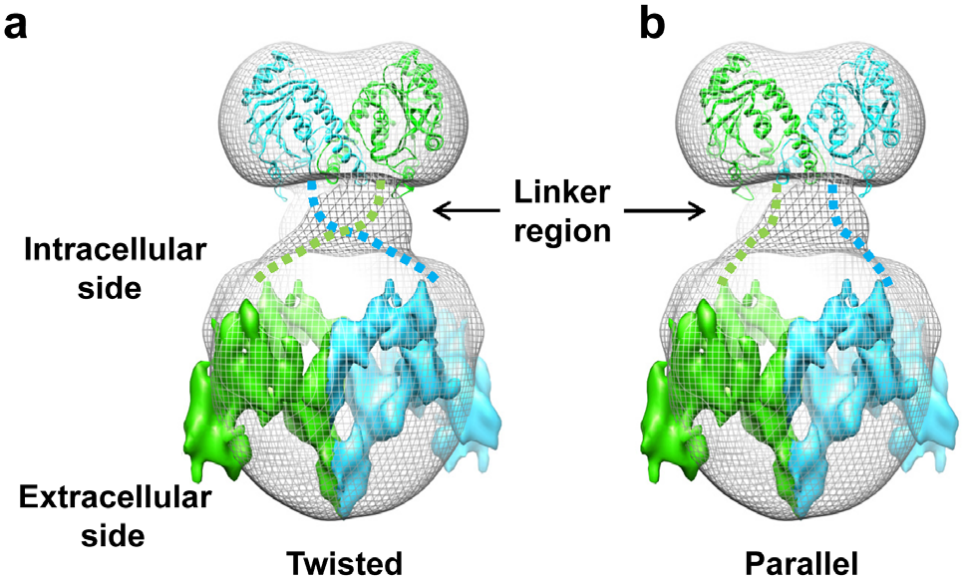
Two possible modes of monomer-monomer association in AE1 dimer. (**a**) The twisted mode of dimerization. (**b**) The parallel mode of dimerization. The crystal structure of cytoplasmic domain (ribbon) and 2D crystal structure of membrane domain (surface) are fitted into the single-particle reconstruction of full-length AE1 dimer (gray mesh) and the two monomers (each consisting of a cytoplasmic domain and a membrane domain) are colored in green or cyan, respectively. The tentative linkers connecting cytoplasmic and membrane domains are depicted as broken lines.

Our single particle reconstruction of full-length AE1 clearly shows that the cytoplasmic and membrane domains are connected through two well separated linker densities (Fig. 3d, right panel). Each linker has a pillar shape and is about 1.5×3 nm in dimension. However, due to the limited resolution of the map, we could not separate the two copies of membrane domains in the large end. For this reason, there are two possible linking topologies, a twisted one or a parallel one, to connect the cytoplasmic and membrane domains between the two ends (Fig. 4).

### Flexibility of Connecting Linkers

**Figure 5:**
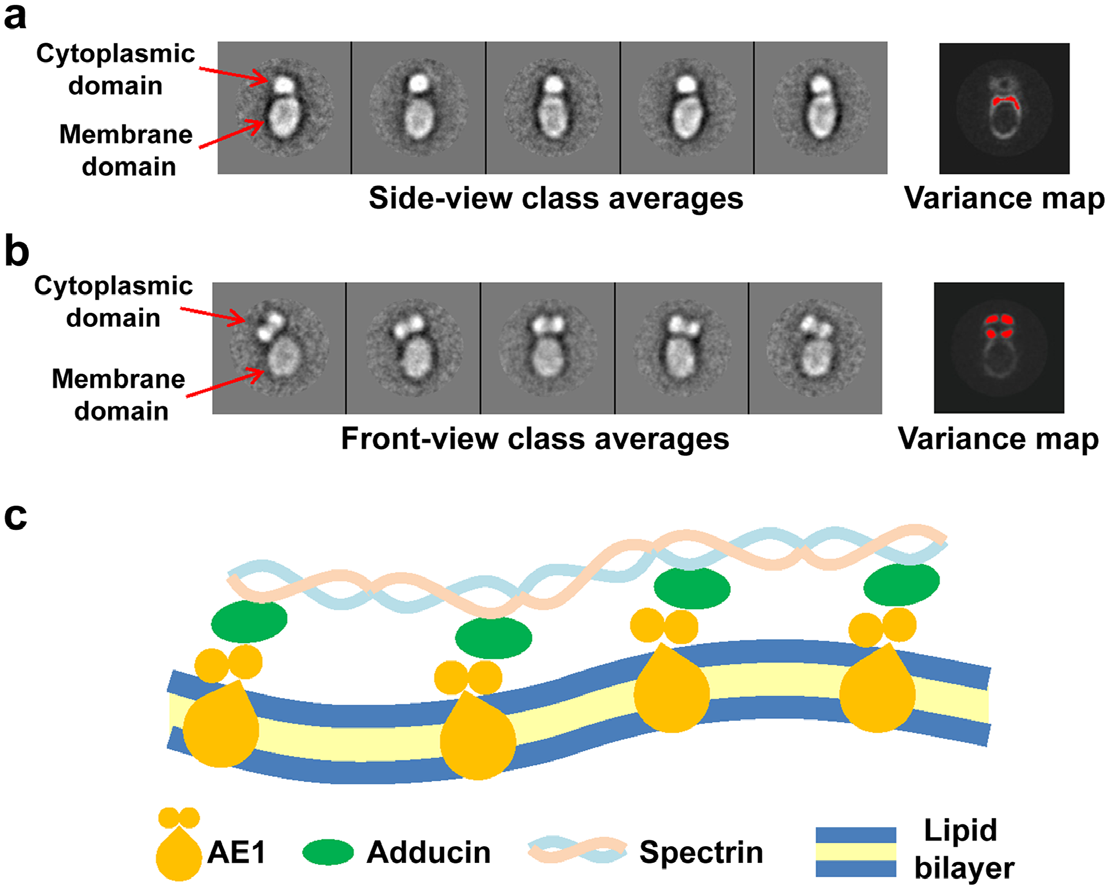
Flexibility of AE1. (**a,b**) Representative class averages and variance maps of the side-view particles (**a**) and the front-view particles (**b**). In the variance maps, the white areas indicate high variance and the areas with statistical significance (>4σ) are highlighted with red color. Note that relative orientation of the projection density corresponding to cytoplasmic domain to that of membrane domain remains uniform in (**a**) but varies in (**b**), suggesting flexibility of the connecting linkers in the sideward direction. The variance maps in (**a**) and (**b**) were calculated from 24 and 58 class averages, respectively. The side length of boxes in (**a**) and (**b**) is 34 nm. (**c**) Mechanistic diagram illustrating AE1 association with the cytoskeleton and how its connector flexibility contributes to erythrocyte shape control. The tilting of the cytoplasmic domain is induced when the erythrocyte membrane is deformed. The connector acts as a pivot between the cytoplasmic and the membrane domains.

To estimate the level of rigidity of the linkers between the cytoplasmic and membrane domains, we selected the front-view and side-view particles (as defined in Fig. 2b,c) and then classified them into sub-classes. In the sub-classes from side-view group, the relative orientations between membrane and cytoplasmic domains are constant (Fig. 5a). In contrast, among the sub-classes from the front-view group, the relative orientations between the two domains vary (Fig. 5b and Movie S1). The distance between cytoplasmic and membrane domains is constant, but the cytoplasmic domain is observed tilting up to 45° along the pivot of the connector region, suggesting significant motions between the domains. The fitting results suggest that the two linkers connecting membrane and cytoplasmic domains are located at the two opposite sides of the side-view particles. Assuming each linker maintains a constant length but hinges around its connecting point with membrane domain, it would allow a tilting motion like that of a teeterboard. The observation that the tilting motion is detected in the front view, but not in the side view, agrees with the teeterboard model of flexibility of the full-length dimeric AE1.

## Discussion

A unique feature of erythrocytes is their ability to squeeze through capillaries of a significantly smaller size than their diameter, and then rapidly restore their original shape following exit into larger blood vessels. Two major mechanisms have been proposed to explain this unique shape and mechanical properties of erythrocytes: the bilayer couple hypothesis and the protein network thesis [40], [41]. Although neither hypothesis fully explains all experimental data, majority of the experimental data are compatible with the protein network hypothesis. The key point of this hypothesis is that the spectrin-actin network anchors to the erythrocyte membrane via rigid AE1 [1], [2], [3], [41]. Wong proposed a dynamic role for AE1 in erythrocyte shape control [42]. It was speculated that two active conformations of AE1, inward-facing and outward-facing, each contracted or relaxed the membrane cytoskeleton by folding and unfolding spectrin, thereby contributing to the erythrocyte shape changes. This speculation has never been confirmed experimentally. Our data suggest that AE1 can participate in mediating erythrocyte shape flexibility via a novel pivot mechanism involving flexible connectors between the membrane and cytoplasmic domains of AE1 (Fig. 5c). This mechanism differs from the one proposed by Wong [42]. and is supported by the observation that the natural mutation in the proposed connector area of AE1 in SAO patients increases erythrocyte rigidity [2], [18].

An earlier study of full-length AE1 by negative staining EM showed different detergents induced different oligomers of AE1, but did not show a 3D structure with distinguishable domains [22]. Our results provide novel information regarding the overall structure of AE1 and provide unprecedented details about the domain organization of a full-length bicarbonate transporter in the SLC4 family. Our structure shows that the cytoplasmic and membrane domains do not occupy stable positions in the protein but instead can move relative to each other. These movements are possible, likely because the connector region is mainly coiled [15]. Our results, when combined with biochemical and crystallography data, secondary structure and topology prediction analyses, and the location of the natural mutations in AE1 affecting the erythrocyte shape control, suggest that the connector region contains approximately 50 amino acid residues [1], [2], [14], [23].

Mohandas and colleagues proposed that the SAO deletion in AE1 induces a conformational change in the cytoplasmic domain, and this change, in turn, leads to a marked increase in the association of this domain with the cytoskeletal network [18]. We hypothesize that the connector region is sufficiently flexible to generate all the necessary conformations of AE1 under mechanical forces induced by erythrocyte movements through narrow capillaries. Such flexibility would also be advantageous for maintaining the integrity of membrane cytoskeleton. In SAO caused by an in frame deletion of aa. 400–408 in the proposed connector region, the erythrocytes have increased rigidity and are oval in shape suggesting that this represents an “experiment of nature” documenting the importance of the connector region in erythrocyte shape flexibility [2], [18].

Two populations of AE1 in the erythrocyte membrane, involved in the cytoskeleton attachment, are currently defined: (1) AE1 tetramer bound to the membrane cytoskeleton via ankyrin, and (2) AE1 dimer linked to the membrane cytoskeleton via adducin. Recent AE1 diffusion measurements in the erythrocyte membrane estimated their near equal relative abundance [43]. Whether a similar pivot mechanism as proposed in the AE1 dimer in our study also exists in tetramers awaits future studies.

## Supporting Information

Movie S1 **Animated view of a series of front-view class averages showing tilting motions of the AE1 cytoplasmic domain relative to the membrane domain.**
(GIF)
