# Correction: Single Particle Electron Microscopy Analysis of the Bovine Anion Exchanger 1 Reveals a Flexible Linker Connecting the Cytoplasmic and Membrane Domains

**DOI:** 10.1371/annotation/5655b604-df69-41d3-8909-31cb45353ed0

**Published:** 2013-10-16

**Authors:** Jiansen Jiang, Nathaniel Magilnick, Kirill Tsirulnikov, Natalia Abuladze, Ivo Atanasov, Peng Ge, Mohandas Narla, Alexander Pushkin, Z. Hong Zhou, Ira Kurtz

There is an error in affiliation for authors Jiansen Jiang, Ivo Atanasov, Peng Ge, Z. Hong Zhou. None of the authors are associated with Structural Computational Biology and Molecular Biophysics Program, Baylor College of Medicine, Houston, Texas. The correct affiliations for all the authors are:

Jiansen Jiang, Peng Ge, Z. Hong Zhou

Department of Microbiology, Immunology and Molecular Genetics, University of

California Los Angeles, Los Angeles, California, United States of America

Jiansen Jiang, Ivo Atanasov, Peng Ge, Z. Hong Zhou

California NanoSystems Institute, University of California Los Angeles, Los

Angeles, California, United States of America

Nathaniel Magilnick, Kirill Tsirulnikov, Natalia Abuladze, Alexander

Pushkin, Ira Kurtz

Department of Medicine, D. Geffen School of Medicine at University of

California Los Angeles, Los Angeles, California, United States of America

Mohandas Narla

New York Blood Centre, New York, New York, United States of America

Ira Kurtz

Brain Research Institute, University of California Los Angeles, Los Angeles,

California, United States of America 

