# Supplementary figures and images for: Single Particle Electron Microscopy Analysis of the Bovine Anion Exchanger 1 Reveals a Flexible Linker Connecting the Cytoplasmic and Membrane Domains
Source: PLoS One. 2013 Feb 5;8(2):e55408. doi: 10.1371/journal.pone.0055408 (PMC3564912; doi:10.1371/journal.pone.0055408)

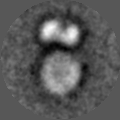

Supplement: Movie S1 — Animated view of a series of front-view class averages showing tilting motions of the AE1 cytoplasmic domain relative to the membrane domain. (GIF) [file pone.0055408.s001.gif]
